# New insights into the phylogeny and historical biogeography of the Lellingeria myosuroides clade (Polypodiaceae)

**DOI:** 10.1371/currents.RRN1197

**Published:** 2010-11-22

**Authors:** Tom A. Ranker, Michael Sundue, Paulo Labiak, Barbara Parris, Germinal Rouhan

**Affiliations:** ^*^University of Hawaii at Manoa; ^†^Tryon Pteridophyte Library at the Pringle Herbarium, The University of Vermont; ^‡^Universidade Federal do Parana, Curitiba-PR, Brazil; ^§^Fern Research Foundation and ^¶^Muséum National d'Histoire Naturelle, UMR CNRS 7205, Paris - France

## Abstract

Grammitid ferns are a well-supported clade of ~900 primarily tropical epiphytic species. Recent phylogenetic studies have found support for a distinctive, geographically diverse group of 24 species referred to as the Lellingeria myosuroides clade and have provided evidence for a variety of phylogenetic relationships within the group, as well as hypotheses of historical processes that have produced current biogeographical patterns. We present new data and analyses that support the following primary conclusions: 1) the L. myosuroides clade is monophyletic and pantropical; 2) that clade is sister to a more species rich clade of entirely Neotropical species (Lellingeria s.s.); 3) we infer two independent dispersal events from the Neotropics to Pacific islands, five independent dispersal events from the Neotropics to the Paleotropics, and two separate dispersal events from mainland tropical America to the West Indies.

## **Introduction**

Grammitid ferns comprise a well-supported clade of ~900 primarily epiphytic, tropical species of the widespread family Polypodiaceae [1] (Parris, unpublished data). One of the long-standing questions in the study of grammitid ferns has been the circumscription of genera, with some authors recognizing a single genus [2] and others up to 24 [3]. The first broad molecular phylogenetic study of grammitids provided new insights and some clarification of generic delimitations. Ranker et al. [4] found that only six of 12 genera for which more than one species was sampled were recovered as monophyletic. Large genera in both the Paleotropics (e.g., *Ctenopteris* Blume ex Kunze s.l., * Grammitis* Sw. s.l.) and the Neotropics (e. g., *Lellingeria* A. R. Sm. & R. C. Moran, *Terpsichore* A. R. Sm.) were found to be polyphyletic. Ranker et al. [4] cited convergent evolution and the use of homoplastic characters by taxonomists to account for the problems in generic circumscription. That study has been followed by more intensive studies of particular grammitid groups (e.g., [5] [6] [7]), some of which have led to nomenclatural changes, including the recognition of new genera [8] [9] [10] [11] [12] [13] [14]. 

      We focus here on the *Lellingeria myosuroides* (Sw.) A.R. Sm. & R.C. Moran group recognized by Smith et al. [15]. Phylogenetic studies of molecular and morphological data provide strong support for the monophyly of this group [16] [5] [4] [7] and its sister relationship to a clade containing all remaining species of *Lellingeria* (the *Lellingeria * s.s. clade of Labiak et al., [5]). The group includes 24 species, which are distinguished from *Lellingeria s.s* by segments with a single unbranched vein, the fertile veins with dark sclerenchyma visible beneath the sporangia, linear hydathodes, and a single sorus per segment (Fig. 1) and will be recognized as a new genus by Labiak [10]. Unlike *Lellingeria s.s.*, which is restricted to the Neotropics, the *L. myosuroides* clade has a pantropical distribution, and is found in the Neotropics (West Indies, Mexico to Bolivia, and Brazil), Hawaiian Islands, French Polynesia, Africa, Madagascar, and La Réunion Island [5]. Estimations of relationships within the *L. myosuroides* clade, including attempts to explain biogeographical patterns, however, have varied across studies. For example, the focus of the work of Geiger et al. [16] was to use molecular phylogenetics to explore the geographical origins of multiple Hawaiian fern lineages, including the Hawaiian endemic *L. saffordii* (Maxon) A. R. Sm. & R. C. Moran (Fig. 1e, 1f). Their results supported a sister relationship between *L. saffordii* and the French Polynesian endemic *L. subcoriacea* (Copel.) A.R. Sm. & R.C. Moran, with that clade being sister to the Neotropical *L. limula* (Christ) A.R. Sm. & R.C. Moran. The clade of those three species was embedded in a larger clade of Neotropical species. Thus, Geiger et al. [16] inferred a single dispersal event from the Neotropics of the common ancestor of the two Pacific species. By contrast, Labiak et al. [5] and Sundue et al. [7] included additional species of *Lellingeria* in their analyses, although the latter did not include *L. subcoriacea*. Both of those studies provided robust support for a sister relationship between the Hawaiian *L. saffordii* and the Mexican *L. hellwigii* (Mickel & Beitel) A.R. Sm. & R.C. Moran. Although the analysis of Labiak et al. [5] could not resolve the exact relationships of * L. subcoriacea*, it was clearly not sister to *L. saffordii* or even the *L. saffordii-L. hellwigii* clade. Thus, they hypothesized two independent dispersal events of the ancestors of *L. saffordii* and *L. subcoriacea* from the Neotropics. 

      We present new evidence of relationships and biogeographical affinities among members of the *L. myosuroides* clade based on phylogenetic analyses of plastid DNA sequences.

**Figure:**
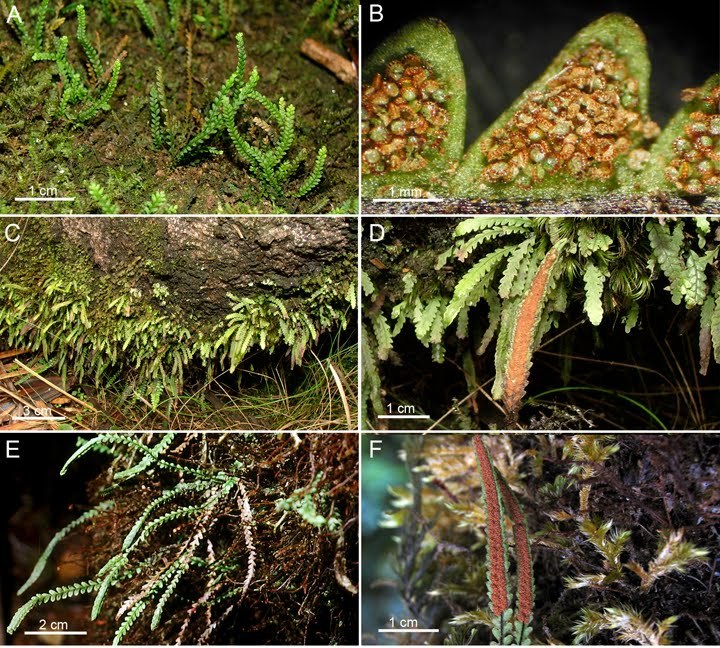
Figure 1. Examples of species in the *Lellingeria myosuroides * clade. A. Habit of *L. pumila * (Brazil). B. Segments and sori of *L. limula*, showing the green spores inside the sporangia (Costa Rica). C. Habit of *L. wittigiana* (Brazil). D. Fertile portion of the lamina in *L. wittigiana * (Brazil). E. Habit of *L. saffordii * (Hawaii). F. Fertile and sterile portion of the lamina in *L. saffordii * (Hawaii). (A, C, and D by P. Labiak; B by R. C. Moran; E and F by T. A. Ranker).  

## **Materials and Methods**

**Taxonomic sampling ** — Outgroup sampling included 18 species (19 accessions) including species of *Lellingeria* s.s., *Melpomene* A. R. Sm. & R. C. Moran*,* the *Terpsichore* *anfractuosa* clade, and the *Terpsichore* *subscabra* clade. These were chosen because they have been recovered as the closest relatives to the * Lellingeria* *myosuroides * clade in previously published studies [4] [5] [7]. The ingroup is represented by 13 species (24 accessions), representing 54% of the known species. Voucher information is listed in Appendix 1. 

***DNA extraction, amplifications and sequencing ***— Genomic DNA was isolated following the protocol detailed in Ranker et al. [17]. The three plastid DNA markers used for the analyses (*atpB*, *rbcL*, and *trnL-trnF*) were amplified by PCR following the protocols detailed in Ranker et al. [17]. All 28 newly obtained consensus sequences have been submitted to GenBank (Appendix 1). 

***Alignment and phylogenetic analyses ***— Consensus sequences were automatically aligned using the program Muscle v3.6 [18], and the resulting alignment was manually checked and revised when necessary. Following alignment, we coded the resulting gaps for the *trnL-trnF* region following the simple coding model as suggested by Simmons and Ochoterena [19], using the program 2xread [20]. Data matrices were constructed using the program ASADO [21] and analyzed using equally weighted maximum parsimony (MP), and Bayesian inference (BI). Maximum parsimony analyses were performed using the program TNT v1.1 [22]. For all MP analyses, heuristic searches were performed with 10,000 parsimony ratchet replicates ([23]; 200 iteration ratchet, the up and down weights set to 5% each), holding 20 trees per ratchet, followed by tree-bisection-reconnection (TBR)-max branch swapping. Support for nodes for the combined data set analysis was calculated by bootstrap analyses (BS), with 1000 replicates doing 10 ratchets per replicate, holding 20 trees per ratchet, and keeping only the strict consensus. The best performing evolutionary model was obtained by the Akaike information criterion (AIC; [24]), using the program jModeltest v.0.1.1 [25]. Bayesian analyses (BI) were performed using the program MrBayes v.3.1.2 [26] [27] on the freely available Bioportal ( www.bioportal.uio.no ). The coded gap characters (for *trnL-trnF*) were included and analyzed separately from the rest of the sequence data, being set to follow the model implemented in MrBayes for binary data: “lset coding = variable”. The partition strategy included the models for each marker, separate codon positions for *atpB *and *rbcL*, and the binary model for the coded gap characters. Three independent runs were started from random trees, consisting of four chains each, one cold and three hot, with the temperature parameter set to 0.2. The analyses were run for 10 million generations, sampling every 1000th generations. To assess whether the Markov chain Monte Carlo (MCMC) reached stationarity after the “burn-in” period, we compared the standard deviation of split frequencies, and by looking at the posterior probability of each clade, as suggested by Huelsenbeck et al. [28], using the online program AWTY [29]. Our data file has been submitted to TreeBase under study number S11022 (http://purl.org/phylo/treebase/phylows/study/TB2:S11022).

  ***Nomenclatural novelties***― The new species mentioned in the text are not intended to be formal proposals. They will be validly published in subsequent articles.

## **Results and Discussion**

      We report here only the results of the analyses of the combined dataset, the resulting phylogenetic trees of which were more resolved and statistically more strongly supported than each tree resulting from the three separate data matrices. The MP analysis recovered three equally parsimonious trees, with L = 628, CI = 0.64, and RI = 0.87. These collapsed at three nodes in a Nelson strict consensus tree. Bootstrap support values (BS) are shown on the strict consensus tree (Fig. 2). Convergence between the three runs of the Bayesian analysis was satisfactorily achieved

**Figure:**
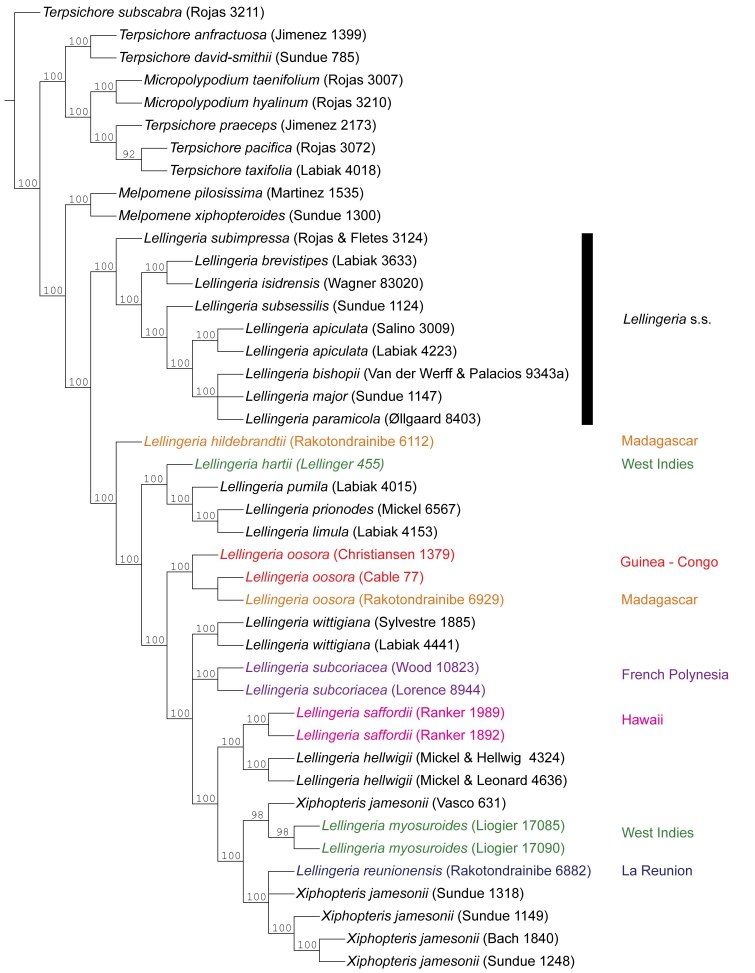
Figure 2. Strict consensus tree from parsimony analysis of the combined dataset. Numbers above branches are bootstrap values. Names in black text are of Neotropical species. Other text colors represent the geographical areas indicated.

according to a plot of the log likelihood (lnL) and analyses performed using AWTY [29]. The 50% majority-rule consensus phylogram from the Bayesian analysis is shown in Fig. 3, which shows the posterior probability (PP) support of the resolved branches. Generally, our results support the relationships discovered by the analyses of Labiak et al. [5] and we will not comment on most of them. As with previous studies, the *L. myosuroides* clade was strongly supported as monophyletic (100 BS, 1.00 PP). Parsimony and Bayesian analyses also strongly supported the sister-taxon status of the Hawaiian *L. saffordii* and the Mexican *L. hellwigii* (100 BS, 1.00 PP). The relationships of the French Polynesian *L. subcoriacea* were not resolved in either analysis but it was clearly not sister to *L. saffordii* or even the *L. saffordii-L. hellwigii* clade. Thus, these results support the hypothesis of Labiak et al. [5] of two independent dispersal events to Pacific islands, probably from the Neotropics, and not the single-dispersal hypothesis of Geiger et al. [16]. Our results also suggest that there were five independent dispersal events from the Neotropics to the Paleotropics, and two separate dispersal events from mainland tropical America to the West Indies. 

**Figure:**
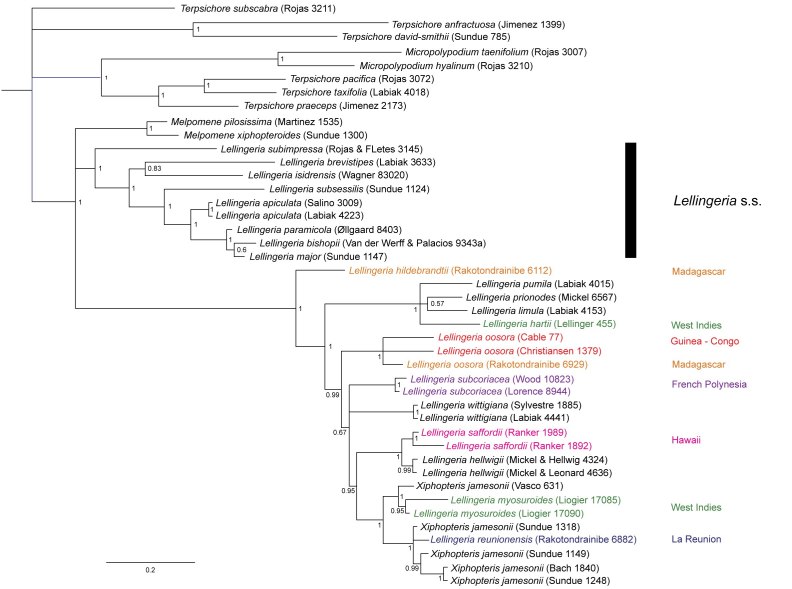
Figure 3. Fifty percent majority-rule consensus phylogram from the Bayesian analysis of the combined dataset. Numbers to the right of or below branches are posterior probabilities. Names in black text are of Neotropical species. Other text colors represent the geographical areas indicated.

      The parsimony analysis also found strong support (100 BS) for a sister-taxon relationship between the *L. saffordii-L. hellwigii* clade and what had been called the *L. myosuroides* clade, as had Labiak et al. [5], but with some significant differences because of the addition of two new accessions. Labiak et al. [5] included data from six South American/Andean accessions labeled *L. myosuroides*, however, recent observations [10] have shown that these Andean specimens should be referred to *Xiphopteris jamesonii* Hook., a species that has been considered as a synonym of *L. myosuroides* by recent authors (e.g., [15] [30]. This species occurs in Central America and the Andes and is morphologically and geographically distinct from *L. myosuroides*, which is restricted to the Antilles. Our dataset included all of the sequence data used by Labiak et al. [5], plus a new accession of true *L. myosuroides* from the Dominican Republic. We also included a new accession from La Réunion Island in the Indian Ocean, which had originally been identified as *L. myosuroides* but will be described soon as a new species, and that is restricted to La Réunion (Parris, unpublished data). We tentatively refer to it as *L. reunionensis* Parris sp. nov. ined. Table 1 presents some of the key differences among *X. jamesonii, L. myosuroides, *and *L. reunionensis*. Our parsimony and Bayesian analyses placed both *L. myosuroides* and *L. reunionensis* within the *X. jamesonii* clade, rendering *X. jamesonii* paraphyletic. *Lellingeria myosuroides* was supported as sister to an accession of *X. jamesonii* (98 BS, 1.00 PP) and *L. reunionensis* was placed with 100 BS and 1.00 PP support as a member of a distinct clade with four other accessions of *X. jamesonii*. One interpretation of this is that *X. jamesonii* is the ancestral taxon for *L. myosuroides* and *L. reunionensis*, both of which diverged from * X. jamesonii* subsequent to independent long-distance dispersals away from the Andes. Alternatively, there could be multiple cryptic species within this clade for which distinctive characters have yet to be discovered. 

     Another interesting pattern is the fact that the *L. myosuroides* clade is geographically highly diverse, having both Neotropical and Paleotropical species, including some extremely localized endemics, whereas its sister clade (*Lellingeria* s.s.) is more species rich but restricted entirely to the Neotropics. We suggest that detailed studies of life history and other attributes may be of value in helping to explain this phenomenon. 

**Table 1. Key characters distinguishing * X. jamesonii, L. myosuroides,* and *L. reunionensis*.**

**Table:** 

	X. jamesonii	L. myosuroides	L. reunionensis
			
leaf length	>10 cm	up to 5 cm	5.3-10.5 cm
lamina segments	deltate, <2x longer than wide or wider than long	linear, >3x longer than wide	deltate, as wide as long or up to 2x longer than wide
lamina segment apex	obtuse to acute	bluntly acute	obtuse
lamina texture	thinly coriaceous	thinly coriaceous	membranaceous to chartaceous
angle of pinnae to rachis	(60-)64-77(-85) degrees	60-75 degrees	(40-)46-55(-60) degrees
lamina indumentum	branched hairs sparse to scattered, 1-5/sq. mm abaxial on rachis and occasional to scattered, <1-5/sq. mm adaxial on rachis; sometimes elsewhere on lamina	branched hairs occasional to sparse <1-2/sq. mm on abaxial surface of lamina, margin & adaxial surface of rachis, sometimes also on adaxial surface of lamina; sparse to scattered 1-5/sq mm on abaxial surface of rachis	sparse w/occasional branched hairs, <1/sq. mm, abaxial on rachis only
petiole indumentum	hairs 2-furcate	hairs often 3-furcate	hairs 1-2-furcate

## **Acknowledgements**

We thank Robbin Moran for use of the L. limula photo.

## **Funding information**

Funding for this project was provided in part by grants to Tom Ranker from the US National Science Foundation (DEB-0344522) and the National Geographic Society. Michael Sundue's research was supported in part by the Burkill Fellowship at the Singapore Botanic Garden.Paulo Labiak's research was partially funded by the Brazilian government (CNPq/PDE n° 201782/2008–1).

## **Competing interests**

The authors have declared that no competing interests exist.

## Appendix 1

**Accession information of species in analyses.  nd = no data**

**Table:** 


Taxon	Provinence	Voucher	atpB	rbcL	TrnL-F
Lellingeria apiculata (Kunze ex Klotzsch) A.R. Sm. & R.C. Moran	Brazil	Labiak 4223 (UPCB)	GU376573	GU387025	GU387215
Lellingeria apiculata (Kunze ex Klotzsch) A.R. Sm. & R.C. Moran	Brazil	Salino 3009 (UC)	AY459480	AY362343	GU476642
Lellingeria bishopii Labiak ined.	Ecuador	Van der Werff & Palacios 9343a (MO)	HQ599531	HQ599522	HQ599516
Lellingeria brevistipes (Mett. ex Kuhn) A.R. Sm. & R.C. Moran	Brazil	Labiak 3633 (UPCB)	GU376575	GU387018	GU387217
Lellingeria hartii (Jenman) A.R. Sm. & R.C. Moran	Dominican Republic	Lellinger 455 (US)	GU376583	GU386985	GU387225
Lellingeria hellwigii (Mickel & Beitel) A.R. Sm. & R.C. Moran	Mexico	Mickel & Leonard 4636 (NY)	GU376584	GU386990	GU387226
Lellingeria hellwigii (Mickel & Beitel) A.R. Sm. & R.C. Moran	Mexico	Mickel & Hellwig 4324 (NY)	HQ599533	HQ599524	HQ599517
Lellingeria hildebrandtii (Hieron.) A.R. Sm. & R.C. Moran	Madagascar	Rakotondrainibe 6112 (P)	GU376585	GU386975	GU387227
Lellingeria isidrensis (Copel.) A.R. Sm. & R.C. Moran	Costa Rica	Wagner 83020 (NY)	GU376586	GU387042	GU387228
Lellingeria limula (Christ) A.R. Sm. & R.C. Moran	Brazil	Labiak 4153 (UPCB)	GU376591	GU387023	GU387232
Lellingeria major (Copel.) A.R. Sm. & R.C. Moran	Ecuador	Sundue 1147 (NY)	GU476766	GU476873	GU476652
Lellingeria myosuroides (Sw.) A. R. Sm. & R. C. Moran	Dominican Republic	Liogier 17090 (NY)	GU376599	GU386988	GU387240
Lellingeria myosuroides (Sw.) A. R. Sm. & R. C. Moran	Dominican Republic	Liogier 17085 (NY)	HQ599534	HQ599525	GU476647
Lellingeria oosora ( Baker) A.R. Sm. & R.C. Moran	Cameroon	Cable 77 (K)	GU376600	GU386972	GU387241
Lellingeria oosora (Baker) A.R. Sm. & R.C. Moran	Rwanda	Christiansen 1379 (US)	nd	nd	GU387242
Lellingeria oosora (Baker) A.R. Sm. & R.C. Moran	Madagascar	Rakotondrainibe 6929 (P)	HQ599535	HQ599527	nd
Lellingeria paramicola Labiak ined.	Ecuador	Øllgaard 8403 (NY)	GU376601	GU387014	GU387244
Lellingeria prionodes (Mickel & Beitel) A.R. Sm. & R.C. Moran	Mexico	Mickel & Beitel 6567 (NY)	GU376605	GU386991	GU387249
Lellingeria pumila Labiak	Brazil	Labiak 4015 (UPCB)	GU376608	GU387022	GU387252
Lellingeria reunionensis Parris ined.	Le Réunion	Rakotondrainibe 6882 (P)	nd	HQ599526	GU476655
Lellingeria saffordii (Maxon) A. R. Sm. & R. C. Moran	Hawaii	Ranker 1892 (COLO)	EF178645	EF178628	EF178662
Lellingeria saffordii (Maxon) A. R. Sm. & R. C. Moran	Hawaii	Ranker 1989 (COLO)	HQ599532	HQ599523	nd
Lellingeria subcoriacea (Copel.) A.R. Sm. & R.C. Moran	Marquesas Islands	Lorence 8944 (PTBG)	EF178646	EF178629	HQ599520
Lellingeria subcoriacea (Copel.) A.R. Sm. & R.C. Moran	Marquesas Islands	Wood 10823 (PTBG)	HQ599537	HQ599529	HQ599519
Lellingeria subimpressa (Copel.) Labiak comb. Ined.	Costa Rica	Rojas & Fletes 3124 (UC)	HQ599536	HQ599528	HQ599518
Lellingeria subsessilis (Baker) A.R. Sm. & R.C. Moran	Ecuador	Sundue 1124 (NY)	GU376612	GU386996	GU387257
Lellingeria wittigiana (F é e) A.R. Sm. & R.C. Moran	Brazil	Labiak 4441 (UPCB)	GU376625	GU387029	GU387270
Lellingeria wittigiana (F é e) A.R. Sm. & R.C. Moran	Brazil	Sylvestre 1885 (UPCB)	GU376624	GU386989	GU387269
Melpomene pilosissima (M. Martens & Galeotti.) A.R. Sm. & R.C. Moran	Mexico	Martinez 1535 (NY)	GU376636	GU386993	GU387281
Melpomene xiphopteroides (Liebm.) A.R. Sm. & R.C. Moran	Colombia	Sundue 1300 (NY)	GU376637	GU387004	GU387282
Micropolypodium hyalinum (Maxon) A. R. Sm.	Costa Rica	Rojas et al. 3210 (CR)	AY459490	AY362344	GU476670
Micropolypodium taenifolium (Jenman) A. R. Sm.	Costa Rica	Rojas 3007 (UC)	AY459491	AY460658	HQ599515
Terpsichore anfractuosa (Kunze ex Klotzsch) B. León & A. R. Sm.	Bolivia	Jimenez 1399 (LPB)	GU476779	GU476833	GU476672
Terpsichore david-smithii(Stolze) A. R. Sm.	Bolivia	Sundue 785 (NY)	GU376639	GU387012	GU387284
Terpsichore pacifica Sundue ined.	Costa Rica	A. Rojas & Mata 3072 (CR)	HQ599530	HQ599521	GU476729
Terpsichore praeceps Sundue & M. Kessler	Bolivia	Jimenez 2173 (UC)	GU476817	GU476839	GU476734
Terpsichore subscabra (Klotzsch) B. León & A.R. Sm.	Costa Rica	Rojas 3221 (CR)	AY459511	AY460677	GU476740
Terpsichore taxifolia (L.) A. R. Sm.	Brazil	Labiak 4018 (UPCB)	GU476800	GU476914	GU476699
Xiphopteris jamesonii Hook.	Colombia	Vasco 631 (NY)	GU376594	GU386968	GU387235
Xiphopteris jamesonii Hook.	Colombia	Sundue 1318 (NY)	GU376598	GU387006	GU387239
Xiphopteris jamesonii Hook.	Ecuador	Sundue 1149 (NY)	GU376596	GU386999	GU387237
Xiphopteris jamesonii Hook.	Colombia	Sundue 1248 (NY)	GU376597	GU387002	GU387238
Xiphopteris jamesonii Hook.	Bolivia	Bach 1840 (LPB)	GU376595	GU386971	GU387236
